# Reactive arthritis following COVID-19: cause for concern

**DOI:** 10.1007/s00167-023-07332-z

**Published:** 2023-02-21

**Authors:** Filippo Migliorini, Jon Karlsson, Nicola Maffulli

**Affiliations:** 1 Department of Orthopaedic, Trauma, and Reconstructive Surgery, RWTH University Hospital, Pauwelsstraße 30, 52074 Aachen, Germany; 2 Department of Orthopaedic and Trauma Surgery, Eifelklinik St. Brigida, 52152 Simmerath, Germany; 3 Department of Orthopaedics, Sahlgrenska University Hospital, Sahlgrenska Academy, Gothenburg University, Gothenburg, Sweden; 4 Department of Medicine, Surgery and Dentistry, University of Salerno, 84081 Baronissi, Italy; 5 School of Pharmacy and Bioengineering, Faculty of Medicine, Keele University, ST4 7QB Stoke On Trent, England; 6 Barts and the London School of Medicine and Dentistry, Centre for Sports and Exercise Medicine, Queen Mary University of London, Mile End Hospital, E1 4DG London, England

**Keywords:** Covid, Pandemic, Reactive arthritis, Musculoskeletal

## Abstract

Low-quality evidence suggests that COVID-19 may trigger reactive arthritis one to four weeks after the infection. Post COVID-19 reactive arthritis resolves within a few days, and no additional treatment is required. Established diagnostic or classification criteria for reactive arthritis are missing, and a deeper understanding of the immune mechanism related to COVID-19 prompt us to further investigate the immunopathogenic mechanisms capable of promoting or contrasting the development of specific rheumatic diseases. Caution should be exerted when managing post-infectious COVID-19 patient with arthralgia.

SARS-CoV-2 Infection (COVID-19) may trigger reactive arthritis at its post-infectious stage through immuno-mediated sequelae, including vasculitis, antiphospholipid antibody syndrome, myositis, and lupus [18]. COVID-19 could promote temporary immunosuppression, activating the immuno-mediated innate response [3, 4, 12, 17, 24, 30]. Though reactive arthritis is commonly triggered by sexually transmitted or gastrointestinal infections, syndromes consistent with reactive arthritis have been observed following Middle East respiratory syndrome, HIV, Dengue, Chickungunya, and Parvovirus B19 [32].

Recently, several case reports originating from India, Pakistan, Italy and the United Kingdom have been published on reactive arthritis following COVID-19 [1, 2, 5–8, 10, 13–16, 19–21, 23, 25, 27–29, 31, 33, 34, 36–40]. The patients, mostly women aged from 30 to 50 years, developed symptoms of reactive arthritis one to four weeks after the infection (mean 22 days), similar to other forms of reactive arthritis. There was no evidence of an association between COVID-19 severity and the onset of reactive arthritis. Most patients recovered within a few days with no additional care or hospitalisation after a diagnosis by exclusion in all patients. Few patients experienced previous severe COVID-19 [14, 16, 21, 27, 33, 34, 37, 38, 40], and only a few required intensive care units (ICU) and mechanical ventilation [14, 27]. Most patients had no history of autoimmunity, inflammatory bowel disease or travel history, and had not been prescribed any new medication. The clinical presentation was variable in terms of imaging, laboratory tests and physical examination. The location and progression of the reactive arthritis were variable. Knees, hands, and ankles were most commonly affected, with the lower back, feet, wrists, and hips less frequently [1, 2, 5–8, 10, 13–16, 19–21, 23, 25, 27–29, 31, 33, 34, 36–40]. Most patients developed asymmetrical oligoarthritis, though some presented with mono-articular reactive arthritis. Similar to other forms of reactive arthritis, tumor, rubor, calor, and dolor were present in most patients [5, 7, 8, 14, 15, 19, 23, 25, 33, 38–40] and, in most cases, no associated extra-articular manifestation was documented, with inconstant nausea, cough, dysgeusia, diarrhoea, and fever. At plain radiography, no bony involvement was evidenced in most patients [2, 10, 14, 19, 21, 25, 27, 33]. Effusion, swelling and oedema were evidenced at magnetic resonance imaging [6, 7, 10, 25, 31, 37] and sonography [8, 14, 21, 25, 29, 37]. Tendinopathy [7, 8, 10, 28], dactylitis [13, 20, 36], and enthesitis of the Achilles tendon [15, 16, 27–29] were common. At aspiration, a mild inflammatory hypercellularity [14, 27] with no evidence of crystal deposition [14, 16, 21, 23, 40] was reported at polarized light microscopy. Serological examination was also variable. Most patients presented higher values of C-reactive protein [1, 2, 6, 8, 10, 15, 16, 21, 28, 29, 33, 34, 40]. Similar to other reactive arthritides, antibodies of rheumatological disorders were occasionally documented: anti-carbamylated protein antibody (ACPA) 1], rheumatoid factor (RF) [1, 38], antinuclear antibodies (ANA) [7], and HLA-B27 [6, 10, 28, 34]. Management was heterogeneous in terms of the type of pharmacotherapy, dosages and length of treatment. Most authors, following the recommendation for the management of peripheral arthritis [9, 13, 22], administered non-steroidal anti-inflammatory drugs (NSAIDs), including celecoxib, naproxen, indomethacin, etoricoxib, and ibuprofen [6, 10, 11, 14–16, 19, 21, 23, 26, 27, 29, 33, 37, 38]. Ten to 80 mg of prednisolone [2, 5, 10, 11, 15, 16, 26, 34, 37] and 4 to 8 mg of methylprednisolone [1, 8] were the most commonly administered steroids. Opioids were also administered orally in two patients [7, 38]. Other less common compounds were sulfasalazine [10, 35], dilaudid [7], neurontin [7], TNF α inhibitor [31], certolizumab [31], leflunomide [26], hydroxychloroquine [26, 28], and methotrexate [1, 28].

Given the intrinsic nature of case reports, it is unclear how generalisable these findings are. High variability in patient characteristics, diagnosis, examinations, and treatment were evident. Established diagnostic or classification criteria for reactive arthritis are missing, and a deeper understanding of the immune mechanism related to COVID-19 may offer a useful opportunity to further investigate the immunopathogenetic mechanisms capable of promoting or contrasting the development of specific rheumatic diseases.

Concluding, low quality evidence suggests that COVID-19 can target the musculoskeletal system causing reactive arthritis at its post-infectious stage. Caution should be used while managing post-infectious COVID-19 patient with arthralgia. It is unclear whether this condition can be prevented, whether previous vaccinations exert a preventive effect, and what the long-term sequelae of COVID-19-related reactive arthritis are.

## Data Availability

The datasets generated during and/or analysed during the current study are available throughout the manuscript.
